# Combined pulmonary and left ventricular support with veno-pulmonary ECMO and impella 5.0 for cardiogenic shock after coronary surgery

**DOI:** 10.1186/s13019-017-0594-4

**Published:** 2017-05-22

**Authors:** Sameh Sayed, Christoph Schimmer, Ina Shade, Rainer Leyh, Ivan Aleksic

**Affiliations:** 1 Department of Cardiothoracic Surgery, Zentrum Operative Medizine, University of Würzburg, Oberdürrbacher Str.6, 97080 Würzburg, Germany; 2 Department of Cardiothoracic Surgery, Assiut University, Assiut, Egypt

**Keywords:** Cardiogenic shock, Extra corporeal membrane oxygenator, Impella 5.0

## Abstract

**Background:**

Mechanical circulatory support is a common practice nowadays in the management of patients after cardiogenic shock due to myocardial infarction. The single or combined use of one or more devices for mechanical support depends not only on the advantage or disadvantage of these devices but also on the timing of use of these devices before the development of multi organ failure. In our case we used more than one tool for mechanical circulatory support during the prolonged and complicated course of our patient with postcardiotomy cardiogenic shock after coronary artery bypass surgery.

**Case Presentation:**

We describe the combined use of Impella 5.0 and veno- pulmonary extra corporeal membrane oxygenation (VP-ECMO) for biventricular failure in a 52 years—old man. He presented with cardiogenic shock after inferior wall ST-elevation myocardial infarction. After emergency coronary artery bypass surgery and failure to wean from extracorporeal circulation we employed V-P ECMO and consecutively Impella 5.0 to manage the primarily failing right and secondarily failing left ventricles.

He remained hemodynamically stable on both Impella 5.0 and VP-ECMO until Heart Mate II left ventricular assist device implantation on the 14^th^ postoperative day. Right sided support was weaned on 66^th^ postoperative day. The patient remained in the intensive care unit for 77 days. During his prolonged stay, he underwent renal replacement therapy and tracheostomy with complete recovery. Six months later, he was successfully heart transplanted and has completed three and half years of unremarkable follow up.

**Conclusions:**

The combined use of VP ECMO and Impella 5.0 is effective in the management of postcardiotomy biventricular failure as a bridge for further mechanical support or heart transplantation.

## Background

Conservative management of cardiogenic shock due to myocardial infarction is associated with mortality rates up to 50% [1]. Early revascularization by percutaneous coronary interventions (PCI) or coronary artery bypass surgery has significantly improved results. However, in profound cardiogenic shock, myocardial revascularization alone may not be sufficient.

Mechanical circulatory support systems like intra-aortic balloon counter pulsation (IABP), extracorporeal membrane oxygenation (ECMO) and ventricular assist devices (VADs) improve outcome especially in case of refractory postcardiotomy low cardiac output syndrome [2]. One or more of these supporting systems may be used in those patients each with its own limitations and complications.

Veno-arterial ECMO has been suggested to be the most useful initial step for urgent stabilization in severe, refractory cardiogenic shock [3]. Short-term ventricular assist devices (VADs) have become a widely accepted treatment option for refractory cardiogenic shock. The Impella 5.0 (Abiomed Inc, Danvers, MA) utilizes minimally invasively placed catheters. It is a 21 F micro-axial pump capable of producing flows up to 5 L/min while positioned across the aortic valve, with the inflow sitting inside the left ventricle and the outflow at the sinotubular junction. It actively unloads the left ventricle and increases coronary perfusion, thereby improving myocardial oxygen supply [4].

## Case presentation

A 52 years old male patient presented with syncope and self-limited ventricular tachycardia in a referring hospital. Inferior wall ST- Elevation myocardial infarction (STEMI) was diagnosed. He developed ventricular fibrillation requiring endotracheal intubation, cardioversion and cardiopulmonary resuscitation for 2 min.

Coronary angiography showed severely diseased left anterior descending, and right coronary arteries (Ejection Fraction < 25% (EF). A right coronary artery angioplasty was unsuccessfully attempted and the patient referred for emergency CABG. An intraaortic balloon pump (IABP) was inserted before surgery and myocardial revascularization was accomplished by bypassing the left internal mammary artery to the left anterior descending coronary artery after thrombendarterectomy and saphenous vein grafting to the right coronary artery. Both grafts had good flow to the target vessels as determined by Doppler flow measurements.

Despite prolonged reperfusion and biventricular pacing weaning from extracorporeal circulation was not possible. Intraoperative transoeosphageal echocardiography (TEE) demonstrated a failing right ventricle, and severely impaired left ventricular function. We decided to support the failing right ventricle by a centrally implanted veno-pulmonary ECMO (V-P ECMO), and left the sternum open due to myocardial edema. Pulmonary artery cannulation was accomplished with two purse string sutures with prolene 5/0 placed in the proximal pulmonary trunk. The PA cannula was tunneled to the left side through a 2 cm stab incision in the 4^th^ intercostal space (midclavicular line) and secured with the purse string sutures. Venous drainage was established through a two stage venous cannula inserted in the right atrium. The cardiopulmonary bypass was transferred to Cardiohelp ECMO circuit (Maquet Cardiovascular, LLC, Wayne, NJ) and the IABP was left in place.

The sternum was kept opened by placing a plastic syringe between the two borders of the sternum. Skin and subcutaneous tissues were covered with sterile dressing which was changed daily in the intensive care unit using strictly sterile technique.

The patient was covered with broad spectrum antibiotic (Cefotriaxone 1 g /8 h), and anticoagulated with heparin keeping the partial thromboplastin time (PTT) at 50–60/s.

In the ICU, the ECMO flow was maintained form 3 to 3.5 L/min and pharmacologic myocardial support was continued to maintain mean arterial blood pressure around 50–60 mmHg and urine output at 0.5 ml/kg/h.

On the postoperative day 2, he developed intractable pulmonary edema, elevated left atrial pressure and hemodynamic instability. TEE showed a dilated left ventricle and persistent poor left and right ventricular contractility (EF =15–20%). We decided to insert Impella Recover LP 5.0 (Abimoed Denver, MA USA) to achieve complete left ventricular unloading and continue the use of V-P ECMO to support the right ventricle. Since the sternum was still open, the Impella was implanted thorough am 8 mm Dacron graft sutured to the ascending aorta The Dacron graft was then subcutaneously tunneled and exteriorized through a separate 2 cm transverse incision in the second right intercostal space just lateral to the sternum. Under TEE control, the Impella 5.0 was then advanced through this Dacron graft into the left ventricle and secured with three ligatures and IABP was removed.

**Fig. 1:**
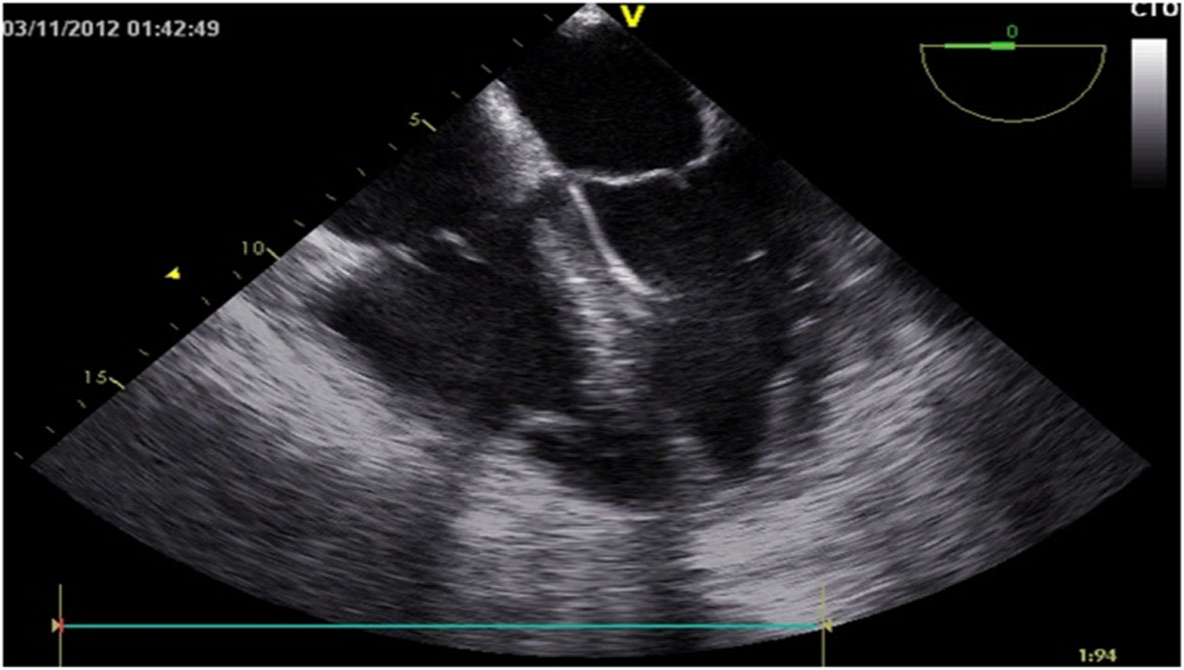
Transesophageal echocardiography in the 3rd post-operative day, showing unloaded Left ventricle after Impella 5.0 repositioning and unloaded right ventricle by VP-ECMO

**Fig. 2:**
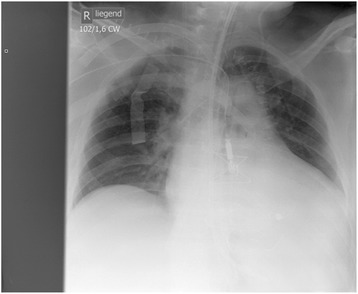
Chest x- ray post-operative day 5, showing biventricular support with V-P ECMO and Impella 5.0

On POD 3, the Impella 5.0 showed low flow alarm; with high driving pressures TEE revealed kinking of the Impella. After repositioning the right side pressure, left side volume overload and pulmonary edema rapidly decreased (Fig. 1). The patient remained hemodynamically stable on biventricular support with V-P ECMO and Impella 5.0 till the 5^th^ postoperative day. When closing the sternum on POD 5, the right atrial two stage cannula was replaced with a long peripheral cannula inserted from the left femoral vein (Fig. 2).

**Fig. 3:**
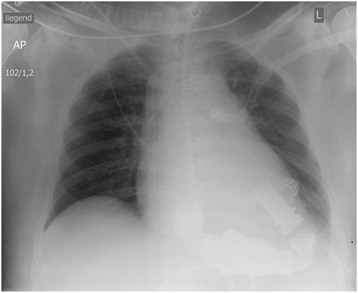
Chest x- ray post-operative day 14 after implantation of Heart Mate implantation with V-P ECMO

Serial TEE follow up showed no improvement of both right and left ventricular contractility (EF 10–15%), necessitating implantation of a permanent LVAD System (Heart mate II- Thoratec Corporation, USA) on the POD 14. Intra operative TEE control showed no right ventricular improvement so we replaced the directly inserted pulmonary cannula of the V-P ECMO with another cannula inserted through a 8 mm Dacron graft sewn to the main pulmonary artery and tunneled subcutaneously and exteriorized through the old incision for future easy removal under local anesthesia by suture closing the graft. (Fig. 3). The patient was consecutively listed for heart transplantation.

After gradual improvement of the right ventricle as demonstrated by serial TEE echocardiography, V-P ECMO could be explanted on POD 66. During this long ICU stay, the patient underwent hemodialysis for renal insufficiency and tracheostomy for prolonged mechanical ventilation with complete recovery. He was transferred to the normal ward on POD 77.

Six months later the patient underwent orthotropic heart transplantation with uneventful postoperative course; he was discharged from the hospital after 7 months of admission in a good condition, and has completed three and half years of uneventful post-transplant follow up to date.

## Discussion

Biventricular failure after myocardial revascularization continues to be associated with high mortality. Urgent resuscitation by short term mechanical assist devices occurs in 1–3% after cardiac surgery. [5] The choice and timing of use of one or more devices depend on the clinical situation as well as the experience of the center.

In our patient we started with an IABP preoperatively, then employed V-P ECMO and added Impella 5.0 to unload the distended left ventricle. The immediate and combined use of different devices of mechanical support was very effective in the management of biventricular failure before the development of multi organ failure. IABP is the most widely used form of mechanical support. However, it lacks active cardiac support and requires a residual level of left ventricular function [6].

In postcardoitomy biventricular failure, VA ECMO has provided adequate end-organ perfusion and it is a more common approach until recovery, however, the imposed afterload and lack of left ventricular decompression causes progressive distention with subsequent more left ventricular failure.

Facing right ventricular failure after myocardial revascularization while the sternum was still opened, we employed a centrally placed V-P ECMO as right ventricular support (RVAD) under the impression that the left ventricle could cope with the remaining function, inotropic therapy and biventricular pacing. We thought that right ventricular support for a few days while the sternum left opened would be sufficient for right ventricular recovery. We used IABP in conjunction with V-P ECMO to reduce left ventricular and pulmonary congestion, however no definite data exist to support its routine use. The employment of V-P ECMO supported the pulmonary circulation for several days, but could not assist the left ventricle which was receiving too much blood from the V-P ECMO. For the purpose of complete short-term unloading of the left ventricle and maintain systemic perfusion we implanted a central Impella 5.0. Total artificial heart could be another option in this scenario but was not employed in our center at that time.

Kawashima has demonstrated that Impella decreases left ventricular end diastolic pressure and pressure volume effective area more effectively than ECMO in the acutely failing heart [7]. These findings suggest that left ventricular mechanical support with Impella may lead to better recovery than ECMO. Based on this hypothesis we tried to support the left ventricle for a few days with Impella 5.0 before proceeding with the permanent LVAD after it become evident that the left ventricle would not recover its function.

The combined use of ECMO and Impella in the successful management of myocarditis has been reported in a few cases [8]. However, there are no reports about the combined use of Impella and V-P ECMO for the management of biventricular failure due to postcardiotomy cardiogenic shock.

This combination, however, has proven to be effective in the temporary management of biventricular failure as a bridge to permanent LVAD with consecutive heart transplantation.

## Conclusions

The decision which device and the timing of employment of each device is a critical point in the management of such complicated cases. The combined use of both Impella 5.0 and V-P ECMO proved to be effective in managing biventricular failure in a patient with cardiogenic shock after coronary bypass surgery.

## Abbreviations

CABG: Coronary artery bypass surgery
ECMO: Extra corporeal membrane oxygenation
EF: Ejection fraction
IABP: Intra aortic balloon pump
ICU: Intensive care unit
LV: Left ventricle
LVAD: Left ventricular assist device
POD: Post operative day
RVAD: Right ventricular assist device
STEMI: ST segment elevation myocardial infarction
TEE: Trans esophageal echocardiography
V-P ECMO: Veno- pulmonary artery extra corporeal membrane oxygenator
